# Diagnostic, Surgical, and Technical Considerations for Lumbar Interbody Fusion in Patients with Osteopenia and Osteoporosis: A Systematic Review

**DOI:** 10.3390/brainsci11020241

**Published:** 2021-02-14

**Authors:** Sauson Soldozy, Samuel R. Montgomery, Danyas Sarathy, Steven Young, Anthony Skaff, Bhargav Desai, Jennifer D. Sokolowski, Faheem A. Sandhu, Jean-Marc Voyadzis, Kaan Yağmurlu, Avery L. Buchholz, Mark E. Shaffrey, Hasan R. Syed

**Affiliations:** 1 Department of Neurological Surgery, University of Virginia Health System, Charlottesville, VA 22903, USA; ss2ah@virginia.edu (S.S.); ds2ky@hscmail.mcc.virginia.edu (D.S.); bd3sb@hscmail.mcc.virginia.edu (B.D.); jde2z@hscmail.mcc.virginia.edu (J.D.S.); kaan_yagmur@yahoo.com (K.Y.); alb8jp@hscmail.mcc.virginia.edu (A.L.B.); mes8c@hscmail.mcc.virginia.edu (M.E.S.); 2 Department of Orthopedic Surgery, University of Virginia Health System, Charlottesville, VA 22903, USA; sm4rb@hscmail.mcc.virginia.edu (S.R.M.J.); as9ey@virginia.edu (A.S.); 3 Department of Surgery, University of Virginia Health System, Charlottesville, VA 22903, USA; sdy4vr@hscmail.mcc.virginia.edu; 4 Department of Neurosurgery, MedStar Georgetown University Hospital, Washington, DC 3800, USA; fasandhu@aol.com (F.A.S.); jmvoyadzis@gmail.com (J.-M.V.)

**Keywords:** lumbar interbody fusion, osteoporosis, osteopenia, minimally invasive surgery, vertebral augmentation, cortical bone trajectory, Hounsfield units

## Abstract

Objective: Osteoporosis is increasing in incidence as the ageing population continues to grow. Decreased bone mineral density poses a challenge for the spine surgeon. In patients requiring lumbar interbody fusion, differences in diagnostics and surgical approaches may be warranted. In this systematic review, the authors examine studies performing lumbar interbody fusion in patients with osteopenia or osteoporosis and suggest avenues for future study. Methods: A systematic literature review of the PubMed and MEDLINE databases was performed for studies published between 1986 and 2020. Studies evaluating diagnostics, surgical approaches, and other technical considerations were included. Results: A total of 13 articles were ultimately selected for qualitative analysis. This includes studies demonstrating the utility of Hounsfield units in diagnosis, a survey of surgical approaches, as well as exploring the use of vertebral augmentation and cortical bone screw trajectory. Conclusions: This systematic review provides a summary of preliminary findings with respect to the use of Hounsfield units as a diagnostic tool, the benefit or lack thereof with respect to minimally invasive approaches, and the question of whether or not cement augmentation or cortical bone trajectory confers benefit in osteoporotic patients undergoing lumbar interbody fusion. While the findings of these studies are promising, the current state of the literature is limited in scope and, for this reason, definitive conclusions cannot be drawn from these data. The authors highlight gaps in the literature and the need for further exploration and study of lumbar interbody fusion in the osteoporotic spine.

## 1. Introduction

Osteoporosis is one of the most common conditions within the elderly population, currently impacting 200 million people worldwide and 54 million in the United States [1]. Postmenopausal white women are at high risk, with 30% having osteoporosis and 16% having osteoporosis of the lumbar spin [2]. Approximately 25% of women older than 70 will experience at least one vertebral body compression fracture (VBCF), with this number rising to more than 50% in women above 80 years old [3,4]. Given the unprecedented rate of growth of the elderly population, a subsequent increase in the incidence of osteoporosis can be expected, and this demographic of patients will make up an increasingly larger proportion of patients seen by spine surgeons. Osteoporosis predisposes patients to deformity and stenosis in addition to fracture, and surgical correction in these patients remains difficult [5,6]. Complications are common and include additional fractures (pedicle and compression), pseudarthroses, instrumentation failure secondary to poor fixation in osteoporotic bone, or spinal disease progression as a result of altered biomechanics [7]. Despite this, evidence remains sparse as to what approaches or strategies should be employed in this patient population.

Examples of these strategies include pharmacologic treatment, using multiple points of fixation in the osteoporotic spine, cement augmentation of pedicle screws, and novel pedicle screw designs targeted at increasing fixation [7]. This study aims to systematically review the current state of the literature with respect to lumbar interbody fusion (LIF) surgery in patients with osteoporosis. In addition to summarizing the findings of these studies, the authors highlight limitations and discuss gaps in the literature that warrant further exploration.

## 2. Methods

**Figure 1:**
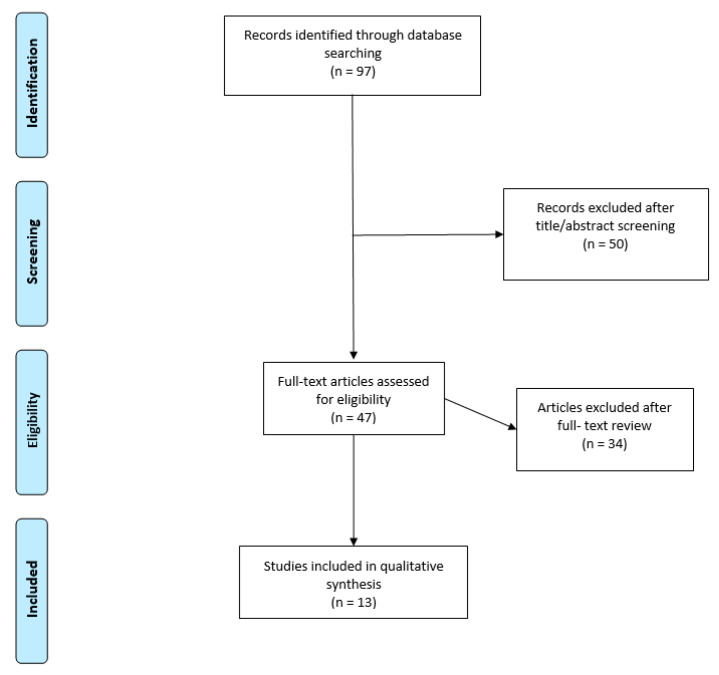
Visualization of study selection. Flow diagram showing process of study selection.

A literature search using PubMed and MEDLINE databases was performed with the search term “lumbar interbody fusion osteoporosis.” This yielded 97 articles. After reviewing the titles and abstracts for relevance, 47 studies were selected. After further review, 13 articles were ultimately selected (Figure 1). Articles evaluating diagnostics, surgical approaches (minimally invasive, anterior, lateral, etc.), and variations in technique (augmentation, screw trajectory) with respect to osteoporotic patients undergoing LIF were included. Review articles, editorials without patient data, and studies assessing pharmacologic interventions were excluded. In addition, articles primarily focusing on complications, such as cage migration, subsidence, and adjacent level vertebral fracture, were excluded.

## 3. Results

A total of 13 articles, summarized in Table 1, were included. We categorized these studies below based on diagnostics, surgical approaches, vertebral augmentation, and cortical bone trajectory. 

### 3.1. Utility of Hounsfield Units

Wagner et al. [8] reported the incidence of undiagnosed osteoporosis via computed tomography (CT) approximation of bone mineral density (BMD) using Hounsfield units (HU) in 128 patients undergoing transforaminal lumbar interbody fusion (TLIF). The average age of patients was 61.5 years, with 77 (60.2%) being male. The authors determined the mean HU of the L4 vertebra on axial CT, and compared the average HU values of those patients with diagnosed lumbar osteoporosis (DEXA BMD < 0.75 g/cm^2^) to those with osteopenia and normal BMD (0.75–0.9 g/cm^2^ and >0.9 g/cm^2^, respectively). Based on 29 patients with DEXA scans, the authors created HU cutoff values for nonosteopenic (HU > 150.1), osteopenic (112.4 < HU < 150.1), and osteoporotic (HU < 112.4) patients based on the 95% CI of mean HU values of these three groups being significantly different. Using these values, the authors found that 63 (49.2%) patients had low BMD of the lumbar vertebrae and 25 (19.5%) had osteoporosis; of those patients with osteoporosis, 10 (40%) were male and 16 (64%) did not have a formal evaluation for osteoporosis. There was no significant difference in BMI, or osteoporosis medication use between the three groups.

Schreiber et al [9] conducted a retrospective chart review to analyze the relationship between HU and successful fusion in 28 patients (52 fusion levels) who underwent lateral transpsoas surgery for LIF. Thirty-eight (73%) of the fusion levels were from female patients, and the average age was 67.3 years. The average construct was 1.9 levels with 12 two-level constructs and 6 three-level constructs. HU analysis was performed on axial CT images in three levels within a vertebral body. The authors assessed union at least 12 weeks postoperatively with HU results blinded; a fusion level was defined to be successful if there was bridging on both coronal and sagittal reformatted CT images. No significant differences in gender, age, BMI, recombinant human bone morphogenetic protein-2 (BMP) usage, preoperative DEXA T score, time to fusion, or the fusion level was found between the nonunion and fused groups. The authors showed that 38 (73.1%) fusion levels were determined to be successfully fused, with mean BMD levels higher compared with the 14 (26.9%) nonunion levels (HU = 203.3 vs. 139.8, respectively, *p* < 0.001). In analyzing union in complete constructs (e.g., all fusion levels having union), the HU values in these 19 patients were significantly higher in the construct, relative to proximal vertebral bodies (HU = 200.4 vs. 133.7, *p* = 0.00001). In the 9 patients with at least one level of nonunion, there was also a significant difference in HU values being higher in the construct as compared to proximal vertebral bodies (HU = 142.3 vs. 107.3, *p* = 0.01). 

A retrospective review by Zou et al [10] studied the relationship between preoperative HU values (axial CT) and the 1-year risk of pedicle screw loosening in 503 patients that underwent lumbar pedicle screw fixation (posterolateral lumbar fusion (PLF) or posterior lumbar interbody fusion (PLIF)) due to degenerative lumbar spine disease. The mean age was 61.2 years, and 315 (62.6%) of patients were female. The authors calculated the HU value by using a single horizontal plane in the middle level of a selected vertebral body and calculated a mean HU value by averaging vertebral levels L1–L4. Outcome assessment was performed 1 year postoperatively by an independent spine surgeon (that did not perform the HU analysis); screw loosening was established when the “clear zone around the pedicle screw exceeded 1 mm.” At 1-year follow-up, 151 (30.0%) patients had screw loosening. Logistic regression analysis demonstrated that independent factors significantly influencing screw loosening included male sex (OR 2.07, *p* = 0.005), length of fixation ≥ 2 levels (OR 3.62, *p* < 0.001), lower mean HU value L1–L4 ≤ 110 (OR 0.977, *p* < 0.002), and fixation to S1 (OR 1.70, *p* = 0.035). Age was not found to be an independent influencing factor (OR 1.034 (0.994–1.076), *p* = 0.1).

### 3.2. Comparing Surgical Approaches

Gu et al. [11] investigated 80 osteoporosis patients (mean age 64.31 years) with lumbar spinal stenosis who underwent either percutaneous transforaminal endoscopic discectomy (PTED) (*n* = 40) or conventional TLIF (*n* = 40). Operation time, blood loss, and hospitalization duration was significantly decreased for patients undergoing PTED compared with TLIF (*p* < 0.05). Preoperative leg and back pain visual analog scale (VAS) scores for the PTED and TLIF groups were not significantly different. Both groups’ postoperative leg and back VAS scores significantly improved (*p* < 0.05); however, leg and back VAS postoperative scores were significantly lower in the PTED group compared with TLIF (*p* < 0.01) at 6 months. Lumbar Japanese Orthopaedic Association (JOA) score was significantly higher and Oswestry disability index (ODI) score was significantly lower in the PTED group (*p* < 0.05). In the PTED group, there was one case of transient nerve stimulation which recovered 2 weeks after the operation. In the TLIF group, there were three cases of nerve injury, two cases of dural bursa rupture, and one case of infection. Overall, the incidence of complications in the PTED group (2.5%) was significantly lower than that in the TLIF group (15%) (*p* < 0.05). All adverse events were completely resolved by the 12th month.

Park et al. [12] assessed repeat decompression and fusion rates after surgery for degenerative lumbar disease at a single level based on different surgical fusion approaches. Procedures included posterolateral fusion and posterior/transforaminal lumbar interbody fusion (P/TLIF) [*n* = 8520 (41.35%) and 12,086 (58.65%), respectively]. The mean patient age was 61.86 years, with 63.68% being women. A total of 170 (0.83%) patients were diagnosed with osteoporosis, and this was not statistically different between the posterolateral fusion and P/TLIF surgery groups (*p* = 0.6717). Cumulative incidence of repeated decompression and posterior fusions was similar in posterolateral fusion (3.15%) and P/TLIF (3.15%). While age, sex, and hospital types were found to significantly affect the risk for repeat surgery, osteoporosis was not shown to confer increased risk of reoperation (*p* = 0.4583, HR = 1.325, 95% CI = 0.630–2.791).

In a retrospective cohort study, Lee et al. [13] explored the outcomes and complications of osteoporotic patients (*n* = 53, mean age 72.1 years) who underwent two-level anterior lumbar interbody fusion (ALIF) with either PLF with open pedicle screw fixation (*n* = 28) or percutaneous pedicle screw fixation (PPF) (*n* = 25). There were no significant differences in age, duration of preoperative symptoms, and follow-up (mean 64.6 months) period between the groups. Mean operation time was significantly shorter for the PPF group than the PLF group for a mean of 240.4 and 313.6 min, respectively (*p* = 0.007). Intraoperative blood loss and postoperative blood loss over two days were also significantly less for the PPF group (*p* = 0.018, 0.021, respectively). A greater number of complications was observed in the ALIF with PLF group (17.9% vs. 8%). However, there were no significant differences in preoperative and postoperative observational measures such as VAS, ODI, and Rolland-Morris disability. No differences in lumbar lordosis or range of motion were demonstrated. Adjacent segment disease occurred at similar rates with no significant difference between the groups (25% vs. 24%). Fusion rates were higher for ALIF with PLF group (85.6%) than with PPF group (76%) at 6 months; however, both arrived at fusion rates of 96.5% and 96% at the last follow-up, respectively.

### 3.3. Vertebral Augmentation

Chandra Vemula et al. [14] published a case series of 25 patients (mean age 61.05 years and female-to-male ratio of 4:1) with lumbar spondylolisthesis (Grades I and II) and osteoporosis undergoing MIS-TLIF with polymethylmethacrylate (PMMA)-augmented pedicle screws. The authors utilized fenestrated cannulated polyaxial screws with fenestrations at the distal end, and bone cement was delivered through bone fillers and adaptor screw complex. No bone cement extravasation occurred intraoperatively, and no patients experienced complications. In addition, there were no instances of screw pullout, and fusion was achieved in all patients. Postoperative VAS scores significantly decreased (*p* < 0.001), and significant improvements in quality of life were noted (*p* < 0.001). 

Wang et al. [15] retrospectively evaluated 88 patients with spondylolisthesis and osteoporosis treated with MIS-TLIF using conventional pedicle screw (CPS; *n* = 52) and fenestrated pedicle screw (FPS; *n* = 36) placement. The FPS is a cannulated polyaxial dual-lead threaded screw with three fenestrations in the distal tip. PMMA bone cement was injected and observed under fluoroscopy to monitor for leakage. Operative time, blood loss, or X-ray time was not significantly different among patients treated with CPS or FPS. All scores including VAS, ODI, and JOA were significantly improved at 3 months postoperatively (*p* < 0.001). There was no significant difference in interbody fusion rates between the two groups. At 12 months, all patients were deemed to achieve satisfactory outcomes. However, patients undergoing FPS placement experienced significantly improved Taillard index scores compared with the CPS group (*p* < 0.001). Cement leakage occurred in 22 of 97 (22.7%) screws, although none of the 13 of 36 (36.1%) experienced symptoms. BMD was found to not be statistically significant between cement leakage-related patients and other patients in the FPS group. 

Cao et al. [16] evaluated the efficacy of pedicle screw with PMMA augmentation in patients with osteoporosis undergoing unilateral TLIF. Osteoporotic patients with degenerative lumbar disease (*n* = 50) were divided into two groups: group 1 (mean age 65.3 years) underwent standard unilateral TLIF and group 2 (mean age 66.9 years) underwent unilateral TLIF with PMMA augmentation. At 2-year follow-up, both Group 1 (*n* = 24) and Group 2 (*n* = 23) showed significant improvements (*p* < 0.05) in VAS back and leg scores, ODI, and JOA with no significant differences between the groups. Disc space height at 10-month and 2-year follow-up was significantly decreased in group 1 (*p* < 0.05). Although the fusion rate was higher in group 2, there was no significant difference between the two groups (*p* = 0.243). In five cases in the PMMA group, leakage into paravertebral soft tissues or paravertebral veins was observed, although this was not clinically significant. In group 1, however, screw loosening occurred in two cases. 

Yun et al. [17] published a series of PMMA augmentation as a salvage procedure via an anterior or posterior approach in 8 osteoporosis patients (mean age 73.4 years) who developed lower back pain after implant failure from their initial operation. Five of the eight patients previously underwent corpectomy, and 4 patients underwent ALIF. All patients underwent vertebral augmentation with PMMA. Mean follow-up between augmentation and the last follow-up was 16 months, with mean follow-up phone call 36.25 months. All patients reported pain relief in the short-term period following PMMA; however, long-term results were reported as unsatisfactory, with 6/8 patients having refused to receive radiologic follow-up due to unsatisfactory clinical results. Objective markers demonstrated surgical success with no loss of correction, fractures, or screw loosening during the follow-up period.

Cyriac et al. [18] published a case report on anterior lumbar interbody fusion (ALIF) with cement augmentation without posterior fixation used to treat low-grade isthmic spondylolisthesis in the setting of idiopathic thoracolumbar scoliosis with secondary degenerative changes. The patient was an osteopenic 66-year-old with multiple comorbidities presenting with 2 years of left radicular leg pain who was found to have a Myerding grade I isthmic spondylolisthesis. After undergoing L5–S1 stand-alone ALIF with cement augmentation without posterior pedicle screw fixation, the patient experienced immediate relief of symptoms without intraoperative complications. The patient remained asymptomatic at 2-year follow-up, with radiographs showing stable fusion of L5–S1 without hardware loosening, vertebral height loss, or subsidence.

### 3.4. Cortical Bone Trajectory 

Liu et al. [19] evaluated surgical outcomes in osteoporotic patients with lumbar degenerative disease after undergoing either midline lumbar interbody fusion (MidLIF) with cortical bone trajectory (CBT) screw fixation compared with TLIF using traditional pedicle screw fixation (TPS). There were 31 patients (mean age 73.42 years) and 32 patients (mean age 74.84 years) in the CBT and TPS groups, respectively. Estimated blood loss and length of hospital stay were not significantly different between CBT and TPS groups (*p* = 0.96 and 0.99, respectively). Radiation exposure (*p* = 0.00) was higher and mean duration of operation (179.68 vs. 143.44 min, *p* = 0.00) was longer in the CBT group compared with the TPS group. Mean VAS scores for back and leg pain improved significantly postoperatively in the CBT and TPS groups (*p* < 0.001). No significant differences in mean VAS leg and back pain scores between the two groups were observed at any time points. The TPS group had a 1.57 times higher rate of persistent symptoms than the CBT group (40.6% vs. 25.8%, *p* = 0.016). In addition, the TPS group had significantly higher rates of screw loosening (28.13% vs. 6.5%, *p* = 0.03) and amount of subsidence (3.01 vs. 2.49 mm, *p* = 0.02) compared with the CBT group. No significant differences were found in other radiological outcomes including gross implant migration or radiologic fusion. One patient has incidental durotomy during surgery in the CBT group, which was repaired without complication. Another patient underwent secondary surgery due to wound infection, and two patients had asymptomatic screw loosening with minimal symptoms requiring no further surgery. In the TPS group, one patient had malpositioning of the L5 pedicle screw that required secondary surgery for screw revision. No neurological deficits were reported in any patients with complications.

A case series by Rieger et al. [20] described a single-level MIS-VLIF (vector lumbar interbody fusion) procedure for spondylodiscitis in 12 patients (mean age 64 years and female-to-male ratio 1:1). Osteoporosis was present in 8 of the 12 patients. The other previously healthy (*n* = 4) patients acquired uncomplicated spondylodiscitis after lumbar sequestrectomy (*n* = 3) or after infiltration therapy for underlying degenerative spinal disease (*n* = 1). Four screws were needed for restoration and preservation of sagittal balance. The authors combined two ipsilateral pedicle screws based on cortical bone trajectories with two crossover laminar screws contralaterally. The tip of the cranial screw lies underneath the upper facet joint and avoids penetration, while the caudal screw penetrates and locks the intervertebral facet joint. The screws are then connected with a single rod. There were no conversions to open surgery during the operation. Postoperative CT scans demonstrated correct instrumentation in all cases, and sagittal balance remained at 6-month follow-up CT scan imaging (*n* = 11). Surgery led to statistically significant improvement in back pain (*p* < 0.001) and improvement in leg pain (*p* < 0.04). Loosening of the ipsilateral cortical pedicle screws was seen on CT in a single patient, which required surgical revision via open procedure. 

## 4. Discussion

Lumbar degenerative disease (LDD) in patients with osteopenia or osteoporosis poses a surgical challenge. In this systematic review, the authors surveyed the current state of LIF in patients with osteopenia or osteoporosis (Table 1).

Three studies involving the use of HU were identified. In the study by Wagner et al., it was noted that many patients older than 50 years old were found to have reduced BMD measurements consistent with at least osteopenia. This suggests that in patients older than 50 undergoing TLIF, BMD approximation from HU measurements under 150 may serve as an alternative screening method to preoperative DEXA scan [8]. Another study correlated HU with successful fusion rates at least 12 weeks postoperatively, suggesting higher BMD may lead to an increased likelihood of fusion at 12 weeks [9]. Zou et al. determined that a low HU value was significantly correlated as an independent factor for postoperative screw loosening in patients 1 year after lumber pedicle screw fixation for the treatment of degenerative lumbar spine disease; however, the authors also suggested other potential influencing factors, such as screw size, postoperative sagittal alignment, and other spinopelvic parameters [10]. These studies suggested that HU may be useful in both presurgical planning and postoperative outcome prediction with respect to fusion rates and screw fixation in osteopenic and osteoporotic patients undergoing LIF. In fact, HU has been used in the evaluation of fatty liver, anemia, and kidney stones [21,22,23]. However, further validation with larger sample sizes is necessary before widespread implementation of HU into clinical practice can be recommended in patients undergoing LIF.

Several studies sought to compare minimally invasive approaches to traditional surgery. Gu et al. [11] suggested that PTED is a safe, if not superior, alternative to traditional TLIF surgery, with reduced complication rates and improved lumbar and leg pain VAS scores in the PTED group. In contrast, Lee et al. [13] found no differences in VAS and ODI scores in patients undergoing two-level ALIF with PLF or PPF. With respect to reduced blood loss, complications, and operation time, ALIF with PPF proves to be superior, although at the expense of a slower fusion rate. This slower fusion rate does not appear to be clinically significant. With respect to posterolateral LIF, PLIF, and TLIF approaches, having osteoporosis does not appear to confer increased risk of fusion failure nor reoperation [12]. As the incidence of osteoporosis rises, so will the need for LIF. Evidently, greater opportunity for prospective comparative studies will emerge to provide further evidence for these findings and parse out differences in surgical approach, if any, as it remains unclear given the inconsistent results of the aforementioned studies.

PMMA-augmented transpedicular fenestrated screw fixation has also been explored in patients with osteoporosis undergoing lumbar surgery. Chandra Vemula et al. [14] demonstrated postoperative improvement in VAS scores and quality of life measures, with no complications such as screw loosening, radiographic lucency, nor bone-cement extravasation. Despite their findings of asymptomatic cement leakage, Wang et al. [15] suggested that MIS-TLIF with FPS bone-cement augmentation provides superior improvements in slip reduction with comparable complication and fusion rates. Cao et al. [16] also observed clinically insignificant PMMA leakage, but experienced reduced disc height space loss and no complications of screw loosening when compared to the standard unilateral TLIF group in their cohort. These preliminary studies suggest thus far that PMMA augmentation has the potential to provide some benefit to osteoporotic patients, with cement leakage being common. While the studies suggest this leakage to be clinically insignificant, this cannot be said for certain as evidence remains limited. Further exploration of its safety is warranted before PMMA augmentation can be recommended as standard of practice.

Cortical bone trajectory (CBT) has been suggested to increase screw and bone interference, which is especially helpful in osteoporotic bone [20]. Liu et al. [19] identified comparable improvements in symptoms among patients receiving traditional pedicle screws compared with CBT screws. The authors demonstrated superiority in CBT fixation with improved lumbar stability, and significantly reduced screw loosening and subsidence rates, suggesting CBT screw fixation may be superior to traditional pedicle screw fixation for lumbar degenerative disease in patients with osteoporosis. Employing a MIS-VLIF, Rieger et al. [20] demonstrated the feasibility of treating lumbar spondylodiscitis in patients with osteoporosis using cortical bony structures for all screw vectors. Notwithstanding, data remain limited with only these two studies having been published to date, exploring CBT screw fixation in the osteoporotic lumbar spine [19,20].

### Limitations and Future Directions

Several limitations exist in our study that should be noted. One drawback inherent to systematic reviews is selection bias, as it is possible that certain articles may have been missed despite our search criteria. Generalizability across studies is limited given the heterogeneity of underlying lumbar disease, study design, populations, procedures, methods, and outcomes. For example, some studies only included patients formally diagnosed with osteoporosis, whereas others also included patients with osteopenia. The limited sample sizes represent another drawback. In addition, not all studies included a control group, and comparisons of outcomes were not uniform across studies.

While definitive conclusions cannot be drawn from this systematic review, it brings attention to the lack of evidence regarding LIF in this patient demographic. This is especially concerning given the gradually increasing size of the ageing population, and therefore increasing incidence of osteoporosis. For this reason, osteoporotic patients will make up a much larger proportion of spine patients undergoing LIF. Given the low number of studies identified in our search, this demonstrates the need for increased validation of the approaches and techniques described above. Therefore, the authors urge spine surgeons to perform further studies in this domain.

At this time, the utilization of HU, PMMA augmentation, or CBT screws cannot be recommended given the lack of strong evidence and high-quality studies exploring these topics in osteoporotic patients undergoing LIF. It remains unclear if osteopenic patients carry the same risk as fully osteoporotic patients. DEXA scanning is not always performed in the recent preoperative period, so it is possible that patients are further along the disease process than previously believed. This represents a potential niche for HU, as the above studies suggest many patients may remain undiagnosed at the time of surgery. Cement augmentation and cortical screw trajectory largely depend on institutional and surgeon preference, warranting comparative studies evaluating differences in outcomes (symptom relief, fusion rate, etc.). No single surgical approach(s) can be definitively stated as superior (or noninferior) as further exploration and validation is required. Furthermore, the question of pharmacologic therapy, not addressed in this study, is another factor that may impact surgical decision making.

Given the above limitations, the authors acknowledge that overarching conclusions cannot be made from this study and that any findings thus far should be treated as preliminary. However, our aim is that this article will provide a snapshot of the current state of LIF in the osteoporotic spine, as well as highlight existing gaps in the literatures that warrant further exploration.

## 5. Conclusions

Osteopenia and osteoporosis will continue to rise in incidence given the growth of the elderly population. This poses a surgical dilemma for the spine surgeon, as it is unclear if variations in surgical approach or technique should be made to compensate for reduced bone mass density in these patients. The findings of this systematic review suggest that Hounsfield units may be an effective clinical tool in the spine surgeon’s arsenal when evaluating these patients preoperatively or predicting outcomes. Additionally, preliminary data demonstrate minimally invasive and percutaneous methods are similar with respect to fusion rates and symptom improvement, with the added benefit of decreased operative time, blood loss, and potentially reduced complication rates in minimally invasive approaches. Vertebral augmentation has potential to reduce screw loosening, although cement leakage is a common occurrence and its clinical significance is unclear. Utilizing cortical bony trajectory pedicle screw fixation may be superior to traditional pedicle screw placement, although this remains unclear. Given the low number of studies and lack of standardized reporting of outcomes, further research is necessary before overarching recommendations can be made in this patient population with respect to lumbar interbody fusion.

## Figures and Tables

**Table 1:** Summary of studies included.

Study	No. of Patients *	Study Type	Focus of Study	Principal Conclusions
Hounsfield Units				
Wagner et al. [8]	(*n* = 128)	Retrospective	The incidence of undiagnosed osteoporosis in lumbar fusion patients.	A large proportion of patients undergoing lumbar fusion surgery have HU evidence of osteoporosis.
Schreiber et al. [9]	(*n* = 28)	Retrospective	The relationship between HU and successful fusion.	Successful lumbar fusion was associated with evidence of higher BMD measured by HU.
Zou et al. [10]	(*n* = 503)	Retrospective	The relationship between preoperative HU and 1-year risk of pedicle screw loosening.	HU was an independent predictor of screw loosening. Lower HU correlated with higher rates of loosening.
Surgical Approaches				
Gu et al. [11]	(*n* = 80)	Randomized Controlled Trial	The safety of PTED in treatment of LSS with osteoporosis.	PTED is safe and effective in treatment of LSS with osteoporosis.
Park et al. [12]	(*n* = 170)	Retrospective	Determine risk factors for repeat decompression and fusion rates after surgery for degenerative lumbar disease in patients who underwent posterolateral fusion vs. T/PLIF.	No difference in rates of repeat decompression or fusion with respect to whether posterolateral fusion or T/PLIF was performed.
Lee et al. [13]	(*n* = 53)	Retrospective	Outcomes in osteoporotic patients following two-level ALIF with either open pedicle screw fixation or PPF.	Two-level ALIF with PPF had fewer minor complications, shorter OR times, and a similar fusion rate compared to open pedicle screw fixation.
Vertebral Augmentation				
Chandra Vemula et al. [14]	(*n* = 25)	Prospective Observational	Evaluate outcomes of MIS-TLIF in osteoporotic patients.	Significant improvement in VAS and ODI score postoperatively with no loosening or screw pullouts.
Wang et al. [15]	(*n* = 88)	Retrospective	Assessing cement-augmented fenestrated pedicle screws in osteoporotic spondylolisthesis patients.	Fenestrated screws had greater reduction of postoperative slip degree without obstructing interbody fusion.
Cao et al. [16]	(*n* = 50)	Randomized Controlled Trial	Evaluate PMMA augmentation in unilateral TLIF in osteoporotic patients.	PMMA augmentation increases fixation stability and decreases disk space height loss without impeding interbody fusion.
Yun et al. [17]	(*n* = 8)	Case Series	Evaluate salvage vertebral augmentation with PMMA following failed LIF.	Salvage with PMMA may offer an alternative way to manage failed interbody fusion.
Cyriac et al. [18]	(*n* = 1)	Case Report	Report a case of using ALIF to treat low-grade isthmic spondylolisthesis due to scoliosis in a patient with secondary degenerative changes.	Stand-alone ALIF with anterior cement augmentation could produce improvement in patients with low-grade isthmic spondylolisthesis in the setting of osteopenia.
Cortical Bone Trajectory				
Liu et al. [19]	(*n* = 31)	Randomized Controlled Trial	Evaluate outcomes in osteoporotic patients undergoing MidLIF with CBT vs. TLIF with TPS.	Patients undergoing MidLIF with CBT screws had similar improvement in clinical symptoms and significantly improved lumbar stability compared to those undergoing TLIF with TPS.
Rieger et al. [20]	(*n* = 8)	Prospective	Evaluate safety of MIS-VLIF in patients with lumbar spondylodiscitis or osteoporosis	MIS-VLIF is feasible and seems to be useful in cases of weak cancellous bone.

* Includes patients with osteoporosis/osteopenia. HU = Hounsfield units; BMD = bone mineral density; PTED = percutaneous transforaminal endoscopic discectomy; LSS = lumbar spinal stenosis; TLIF = transforaminal lumbar interbody fusion; PLIF = posterior lumbar interbody fusion; ALIF = anterior lumbar interbody fusion; PPF = percutaneous pedicle screw fixation; MIS-TLIF = minimally invasive transforaminal lumbar interbody fusion; VAS = visual analog scale; ODI = oswestry disability index; PMMA = polymethyl methacrylate; CBT = cortical bone trajectory; MidLIF = midline lumbar interbody fusion; TPS = traditional pedicle screw fixation; MIS-VLIF = minimally invasive vector lumbar interbody fusion.
